# Enhancing robustness of interdependent network under recovery based on a two-layer-protection strategy

**DOI:** 10.1038/s41598-017-13063-2

**Published:** 2017-10-06

**Authors:** Maoguo Gong, Yixing Wang, Shanfeng Wang, Wenfeng Liu

**Affiliations:** Key Lab of Intelligent Perception and Image Understanding of Ministry of Education, International Research Center for Intelligent Perception and Computation, Xidian University, Xi’an, Shaanxi Province 710071 China

## Abstract

The robustness of coupled networks has attracted great attention recently, because the spread of failures from one network to its coupled network makes the coupled networks more vulnerable. Most existing achievements mainly focused on the integrity properties of coupled networks. However, failures also exist when networks are being reconstructed. Moreover, existing node-protection methods which aim to enhance the robustness of coupled networks only protect the influential nodes in one layer. In this paper, firstly, a two-layer-protection strategy is proposed to enhance the robustness of coupled networks under their reconstruction. Secondly, we adopt five strategies based on different centralities to select influential nodes, and propose a two-layer vision for each of them. Lastly, experiments on three different coupled networks show that by applying the two-layer-protection strategy, the robustness of coupled networks can be enhanced more efficiently compared with other methods which only protect nodes in one layer.

## Introduction

Recently, the property, function, and their relations of complex networks have attracted much attention. Complex networks are used to represent the real systems in the form of graphs which consist of nodes and edges^1^. Nodes are used to represent the entities of real systems, and edges represent information interactions or other relations among entities. Studies on the nature of complex networks have received abundant achievements^2–5^, such as acquaintance networks (in the field of social networks)^6^, neural networks (in the field of biology)^5^, and the Worldwide Web (in the field of technology)^7^. Nowadays, with the significant progress made by people in the fields of transportation, electronic, and computer science, networks have become more and more complex^8^, which means that there is a high risk when complex networks face attacks or failures. The above phenomenon explains why the robustness of complex networks^9,10^ has become the research focus.

The robustness of networks measures the remaining structural integrity of networks when unpredictable changes such as attacks or failures occur on them. To be more detailed, the robustness of networks is estimated in the first time by considering the critical component of networks when they are damaged completely^9^. But in the real world, the situation that a network is completely destroyed is relatively rare, and the more common situation is that a network is partly destroyed but will still have some functional components. Schneider’s model^11^ has become the mainstream criterion of the network robustness. The measure *R*_*n*_ considers the sum of the remaining fraction of the largest connected component in every iteration when nodes are gradually removed. Then, the criterion can be adopted in every possible condition of networks facing failures or targeted attacks. *R*_*n*_ can be computed as ${R}_{n}=\frac{1}{N}\sum _{p=1}^{N}S(p)$, where p is the number of removed nodes, *s*(*p*) represents the fraction of the largest connected component when *p* nodes fail. The $1/N$ factor makes it convenient to compare the robustness of networks with different sizes.

In recent years, networks have become more dependent on others. In the real world, many systems have their coupled networks. Different infrastructures are coupled with others, even coupled together. For example, power stations are coupled with the water supply system, transportation, and the Internet. The functionality of the Internet not only relies on itself, but also on the power system. The Internet system cannot work normally without power, which means if power stations are broken down, the water supply, transportation system, etc., would also face a substantial risk of cascade failures at the same time. The electrical blackout in Italy in 2003 is a real-world instance to study the process of the cascade failure: firstly, damages on power stations caused nodes failure in the Internet communication network, which led to a breakdown of power stations in return. In the end, the entire system disintegrated^12–14^. Study of this coupling property can help people to learn more about how the modern system works. A recent research proposed a framework to study the process of cascade failures of coupled networks^14^. It shows that due to the coupling property, networks become extremely fragile facing random failures, and in this situation, even a small failure in one network would trigger a cascade breakup in the whole system. For instance, assuming a system coupled by network A and B, failures of some nodes in network A would lead to damage of nodes’ functionality in network B, and the damage would spread to A in return^15,16^. This circulatory failure may cause a complete crash of the entire coupled networks. In the end, only the largest component (largest connected cluster) of the survived nodes are still in function^12,13^.

Recently, more and more attentions have been paid on the robustness of coupled networks. Many novel models have been proposed to improve the robustness of coupled networks when the functionalities of them are damaged. In Schneider’s research, by selecting autonomous nodes, the robustness of coupled networks can be tremendously enhanced^17,18^. The autonomous nodes do not lose their functionality when their coupled nodes suffer from damages. Because of the partial-independent property, the degree of coupling decreases. And in the same study, the robustness of coupling networks is greatly increased by setting 10% autonomous nodes. Compared with single-layer networks, coupled networks are extremely vulnerable to failures^15^. Huang *et al*. showed that only protecting the high-degree nodes cannot efficiently improve the robustness of coupled networks^19^. Huang developed a mathematical framework to solve targeted-attack problems by mapping them to random-attack problems^19^. Besides, there is another model of cascade failures in coupled networks developed by Zio^20^. In their works, two parameters are used to simulate the cascading failure process: *L*_*cr*_ indicates the critical load, and S represents the average cascade size. They can together identify cascade-safe regions for interdependent networks and with which the robustness of the system is enhanced. According to ref.^21^, by adjusting dynamically the capacity of overload nodes, without changing the price of the coupling system, they proposed a strategy to protect the overload nodes from failures. There are also some protection-based strategies which can improve the robustness of coupling networks by protecting nodes, such as Degree centrality^22^, Betweenness centrality^22^, LeaderRank centrality^23^, Local centrality^24^.

As for applications in the real world, what should be done first to rescue the functionality of a damaged system is to gradually reconstruct the damaged entities^25^. Reconstructing a damaged system can be regarded as an inverse procedure of attacking, and it can be modeled as a process in which the damaged nodes are gradually revived. In the recovery processes, nodes in a network can be triggered to work normally if their coupled nodes in another network have been revived. The cascading failure would also occur in the recovery procedure and damage the system as well as in the attacking situation. When it happens, the system should be revived as soon as possible before things become any worse. Reactive all nodes in the same time would be a solution to recover the system. But in real world, it would cost a lot to simultaneously fix all the nodes. In this paper, we adopt a targeted-recover strategy which recover the high-degree nodes gradually^25–27^. Ma *et al*. proposed a model to enhance the robustness of coupled networks under their recoveries^28^. They found that by protecting influential nodes, the robustness of coupled networks can be greatly improved under their target recoveries. In their work, they compared the robustness by protecting nodes based on different strategies. Concretely, by protecting influential nodes selected by different standard (Random, Degree, Betweenness, LeaderRank, Local, PageRank^29^), the robustness of coupled network can be greatly improved.

Methods which are mentioned above have been proved to be efficient ways to enhance the coupling systems. But they only focus on protecting nodes in one layer (one network in the coupled system) of a coupled system under attacks. It turns out that in the real world, one network is as important as its coupled network because they are at same risks of failure and the system cannot operate if one of them lose its functionality^12,15,30^. Besides, the status of a node in one network cannot be used to measure how important it is in the whole coupled networks. There are “global” nodes which are easily to spread the damage to their neighbors or to their coupled nodes. For example, when reconstructing the Power-Internet system, there are nodes in Power network which are vulnerable and in the meantime their coupled nodes play an important role in the Internet network. When these nodes are shut down because of failures, it would damage the Internet networks. Based on the above discussion, it is reasonable to take the whole coupled system into consideration instead of only one network of the system, and to protect the nodes that are influential for the whole coupled networks. A way is to temporarily fuse the two coupled networks into one.

In this paper, we propose a two-layer-protection (TLP) method to enhance the robustness of coupled networks under recoveries, in which we protect influential nodes of the entire two-layer coupled networks instead of those only in one of the network in the coupled system. Firstly, we generate a pair of totally damaged coupled networks, and study the process of cascade failure under recoveries. Secondly, for five different strategies, we propose a technique based on a two-layer strategy to select influential nodes in coupled networks respectively. Finally, experiments on artificial networks and real-world networks shows the performance of the proposed method.

## Results

The performance of proposed strategy in enhancing the robustness of networks under recoveries is given. And the model is tested on the following three damaged coupling networks. Nodes in the tested networks are coupled with each using the model in refs^14,15^. Each node in one network is randomly coupled with the one in the other network and in this way, a coupling system becomes a pair of networks.

### ER-ER system

Networks that have the random-connected property have been widely studied. These “random connecting” networks represent the topology of a lot of traditional networks and are often modeled as Erdő-Rényi(ER) random networks^31^. In ER random network, two nodes are connected with probability p, and the average degree $\mathop{k}\limits^{\_}$ is calculated as p * N, where N is the total number of nodes. The proposed strategy are tested on a completely damaged coupling ER-ER system (coupled by a totally damaged ER network with N = 5000 and $\bar{K}=2$ and an ER network with N = 5000 and $\bar{K}=2$) to simulate the traditional graphs.

### SF-ER system

Modern systems tend to have a scale-free (SF) property, such as social networks (including collaboration networks), interbank payment networks^32,33^, Semantic networks^34^. A scale-free network is a network whose degree follows a power law distribution. That is, the fraction P(k) of nodes in the network having k connections to other nodes goes for large values of k as $P(K) \sim {k}^{-\gamma }$, where *γ* is an exponential parameter whose value is typically in the range $2 < \gamma  < 3$. Not all the systems are coupled by networks with same property in the real world, so it is important to analyze composite coupled networks. The proposed strategy is tested on a completely damaged coupling SF-ER system(coupled by a totally damaged ER network with N = 5000 and $\bar{K}=2$ and a SF network with N = 5000 and *γ* = 2.4.

### SF-Power system

More and more modern networks have a scale free property and in the meantime, they also have a modular structure with which some nodes link densely with each other but connect sparsely with other nodes of the network. A U.S. Power Grid network (power) with N = 4941 nodes and M = 6954 edges have both scale-free property and modular structure. In this paper, the completely damaged power network is coupled with a SF network with N = 4919 and *γ* = 2.2 to simulate a real-world coupling system.

In the experiments, we compute the *R*_*rc*_ and *R*_*rl*_ of three different kinds of coupled networks, which are listed above. The reason why we choose *R*_*rc*_ and *R*_*rl*_ as criterions is that traditional measurement like Rn only consider half of a coupled system and cannot show how well a two-layer strategy performs. *R*_*rc*_ and *R*_*rl*_ are computed as follows:

### R_rc_

Based on the model of Ma^28^, in a system coupled by networks A and B, the cascade failures under recoveries when a fraction of nodes *p* in network A are recovered can be modeled as equations (1)

1 $$\begin{array}{c}{{\rm{\Theta }}}_{n}^{r}(p)=p,\\ {{\rm{\Delta }}}_{n}^{r}(p)={q}_{{\rm{\Delta }},n}^{r}{S}_{A}^{r}({{\rm{\Theta }}}_{n}^{r}(p))p,\\ {{\rm{\Theta }}}_{n}^{r}(p)={q}_{{\rm{\Theta }},n}{S}_{B}^{r}({{\rm{\Delta }}}_{n-1}^{r}(p))p\end{array}$$

when the fraction of nodes *p* in network A are recovered, ${{\rm{\Theta }}}_{n}^{r}(p)$(${{\rm{\Delta }}}_{n}^{r}(p)$) represents the fraction of surviving nodes in network A(B) at the (n-1)th failing process. ${S}_{A}^{r}({{\rm{\Theta }}}_{n}^{r}(p))$(${S}_{B}^{r}({{\rm{\Delta }}}_{n}^{r}(p))$) is the proportion of nodes of the largest part in the network A(B), and ${q}_{{\rm{\Theta }},n}$(${q}_{{\rm{\Delta }},n}$) is the fraction of nodes in network A(B) which are coupled with the recovered nodes in network B(A). The cascade failure will recursively occur until the equations (2) are satisfied.

2 $$\begin{array}{c}{{\rm{\Theta }}}_{n+1}^{r}(p)={{\rm{\Theta }}}_{n}^{r}(p),\\ {{\rm{\Delta }}}_{n+1}^{r}(p)={{\rm{\Delta }}}_{n}^{r}(p)\end{array}$$

For a system coupled by network A and B, the integrity depends on not only the function nodes in network A, but also the fraction of remaining nodes of network B. In Ma’s model^28^, an index *R*_*rc*_ is proposed to judge the recovery robustness with the functionality integrity of both networks are considered. Equation (3) shows the computation of *R*_*rc*_:

3 $${R}_{rc}=\frac{1}{N}\sum _{p=1/N}^{1}{f}_{rc}(p)=\frac{1}{N}\sum _{p=1/N}^{1}p{S}_{A}^{r}{({{\rm{\Theta }}}^{r}(p))}^{\lambda }{(p{S}_{B}^{r}({{\rm{\Delta }}}^{r}(p)))}^{1-\lambda }=\frac{1}{N}{\sum _{p=1/N}^{1}p{({S}_{A}^{r}({{\rm{\Theta }}}^{r}(p)))}^{\lambda }({S}_{B}^{r}({{\rm{\Delta }}}^{r}(p)))}^{1-\lambda }$$

where ${f}_{r{\rm{c}}}(p)$ represents the integrity of the whole system when the fraction of nodes $p$ in network A have been recovered. ${{\rm{\Theta }}}^{r}(p)$ and ${{\rm{\Delta }}}^{r}(p)$ can be calculated by equation (1). λ is a mixing parameter ranging from 0 to 1 which refer the situation that robustness is determined only by network A(B).

Results of *R*_*rc*_ are given in Table 1 (as the column ‘No’): among the original systems, the ER-ER system performs best against cascading failures and shows a highest *R*_*rc*_ under recoveries in the three systems. The reason that the original ER-ER system is robust is that its nodes are connected randomly, while SF-ER and power-SF are brittle because of the scale-free property. Scale-free property of network means that some nodes of it are linked densely while some are connected sparsely. When a node in a closely connected component failed, a severe cascading failure will occur.

**Table 1:** Comparisons of *R*_*rc*_ (λ = 0.5) between existing methods and two-layer protection based on five strategies.

	No	Degree	Local	Betweenness	Comm	Random
ER-ER						
single-layer	0.3744	0.3997	0.3911	0.3988	0.3928	0.3918
two-layer	0.3744	0.4245	0.4147	0.421	0.4143	0.412
SF-ER						
single-layer	0.3485	0.3804	0.3837	0.3842	0.3725	0.3618
two-layer	0.3485	0.4652	0.4164	0.4614	0.4425	0.389
SF-Power						
single-layer	0.403	0.426	0.4267	0.4397	0.4172	0.42
two-layer	0.403	0.4478	0.4273	0.4626	0.4341	0.4388

Then, five strategies are adopted to protect 5% influential nodes, and compare the performance between them and the proposed two-layer vision of them under their recoveries. The corresponding results are given in Table 1 and as it shows, performances are greatly improved since a small fraction of nodes (5%) are under protection, especially for the Betweenness protection and Degree protection strategies. To be more detailed, for the tested ER-ER coupled networks, under the Betweenness strategy, the *R*_*rc*_ can achieve 0.3988 from the original 0.3744, whose improvement reach 6.52%, 10.24% for ER-SR system (from 0.3485 to 0.3842), and 9.11% for SF-Power system (from 0.4030 to 0.4397). But with a two-layer Degree strategy, improvement can reach 12.45% for the ER-ER system (from 0.3744 to 0.4210), 32.40% for the SF-ER system (from 0.3485 to 0.4614), and 14.80% for the SF-Power system (from 0.4030 to 0.4626). Comparing to single strategies, the proposed two-layer method can achieve considerable improvements which are 5.57% for ER-ER system, 20.09% for SF-ER system, and 5.68% for the SF-Power system. This is because influential nodes which are selected by the proposed strategy are more “global” than those chosen by the traditional ways since cascading failures occur on both sides of a system. And in this way, every node can become a “global” node and the status of a “global” node can be used to measure how important it is in the entire coupled networks. It is notable that the improvement on the ER-ER system is much less than the SF-ER system. This is because nodes in ER-ER networks are connected randomly, which makes every node in the system has the same property. As Table 1 shows, the improvement on the SF-Power is also less than on the SF-ER system. This is because the SF network (in the paper LFR network is used as an example) and the Power network are more likely to have their own features. Performance on SF-ER and SF-Power system can be greatly improved because in the two-layer strategy, nodes are selected to be protected according to their values in the whole coupled system instead of only in on network. From Table 1 we can conclude that, the two-layer method can improve the robustness of a coupled system more efficiently and have a 10.45% improvement on average compared to the single layer protection.

**Figure 1:**
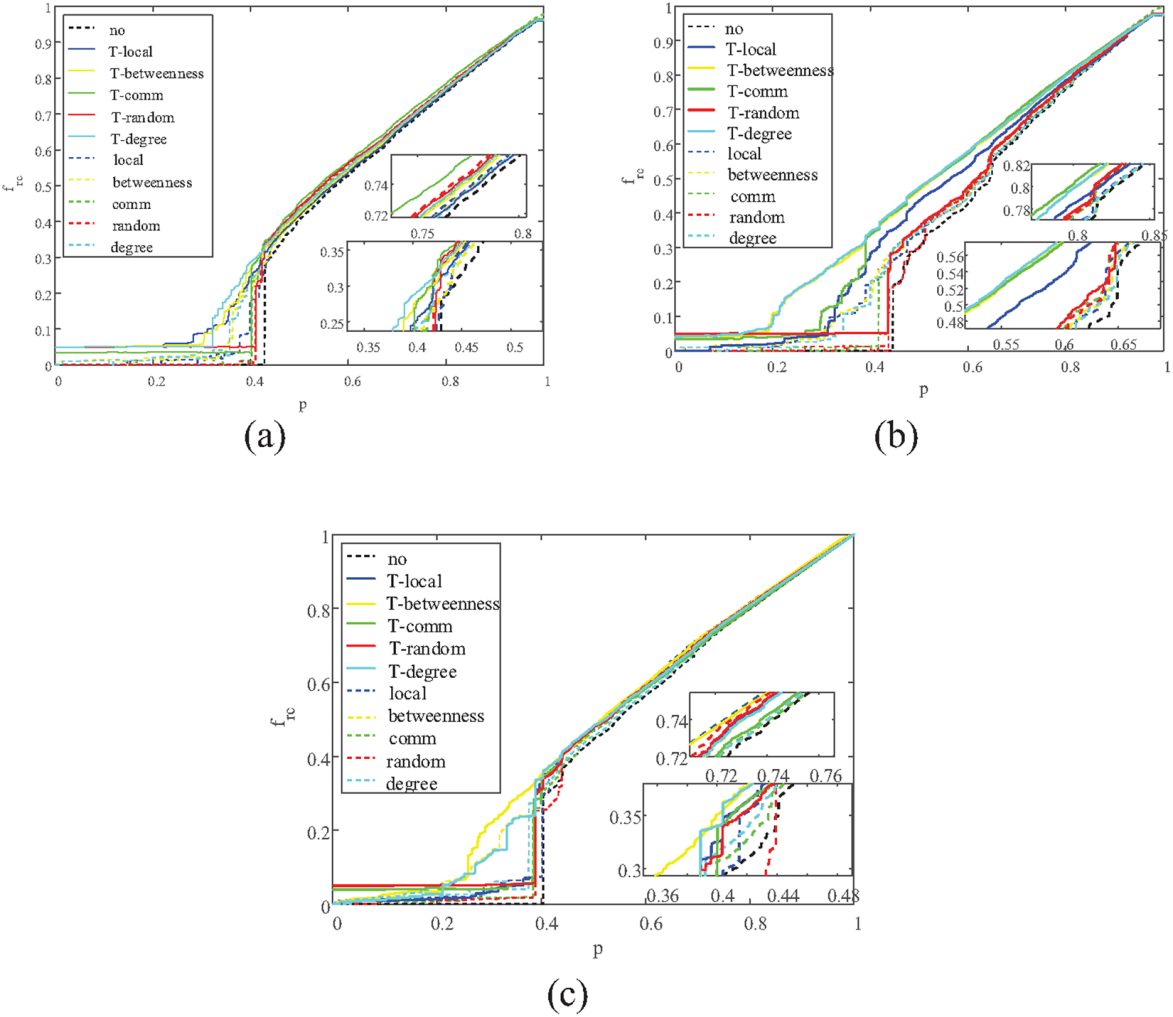
Illustration of the remaining fraction of nodes in each iteration $({f}_{rc}(p))$. When fraction p of nodes are recovered. Curves of different color represent different protect strategies, and dashed lines represent the method of single-layer protection respect to a specific strategy while solid lines are their two-layer version. (**a**) Correlation between ${f}_{rc}(p)$ and recovered nodes p on ER-ER system. (**b**) Correlation between ${f}_{rc}(p)$ and recovered nodes p on SF-ER system. (**c**) Correlation between ${f}_{rc}(p)$ and recovered nodes p on Power-ER system.

In order to analyze the variation of *R*_*rc*_ and compare the performance between single-layer protection and two-layer protection in the recover process on the three types of systems, the variations of functional fraction of nodes in the largest connected component ${f}_{r{\rm{c}}}(p)$ are analyzed when a fraction of p nodes are revived. The simulations of *R*_*rc*_ are shown in Fig. 1. The illustration shows that the two-layer protection can transform coupled networks from a first order phase transition into a second order phase transition. But there are two exceptions: nodes in ER-ER network connect randomly, which means methods based on modular structure such as two-layer Comm cannot performance well on it, on the SF-Power network, T-Comm doesn’t perform well because the SF(in this paper LFR are used as an example) network and Power network have their own structures which would affect the two-layer strategies. It is notable that with the two-layer protection, the ER-ER networks, the SF-ER networks and the SF-Power networks begin to recover their functionalities when around 30%, 20%, and 25% nodes are recovered respectively, comparing to 36%, 30% and 38% with single-layer protection.

**Figure 2:**
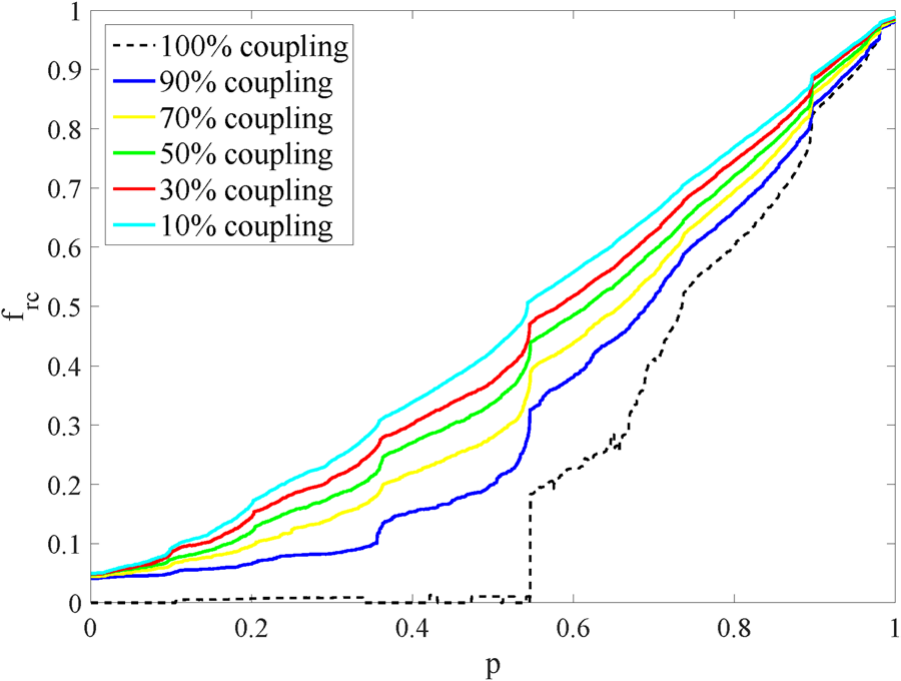
Illustration of the remaining fraction of nodes in each iteration $({f}_{rc}(p))$. When fraction p of nodes are recovered tested on the ER-SF network. Curves of different color represent different coupling rate. In order to show the difference clearly, nodes are coupled under a same role when they are recovered and for the same reason only curves under the two-layer-protection are showed in the illustration.

It is reasonable to conclude that, under every centrality strategy, networks with two-layer protection always begin to recover their functionalities before those with single-layer protection. The results of the two-layer betweenness strategy on the ER-SF networks with different coupling rates are shown in Fig. 2, and the values of *R*_*rc*_ are shown in Table 2. Figure 2 shows that a network which is strongly coupled with another network is more fragile to failures. This is because failures are easy to spread from one network to another in a strongly coupled system. To highlight the difference between networks with different coupling rates, networks are coupled under a same role in the recover process. The illustration shows that networks with lower coupling rate always begin to recover themselves before those with high coupling rate. Besides, from Table 2 we know that, networks with lower coupling rate are more robust than those with higher coupling rate.

**Table 2:** Comparisons of ${R}_{rc}(\lambda =0.5)$ between SF-ER networks with different coupling rates under the two-layer Betweenness strategy.

coupling rate	No	90%	70%	50%	30%	10%
two-layer-strategy	0.2537	0.3457	0.3859	0.4157	0.4401	0.4655

### R_rl_

Average inverse geodesic length^35^ is widely used to analyze the robustness of networks, which can be computed as

4 $${\rm{L}}=\frac{1}{{N}^{2}}\sum _{i=1}^{N}\sum _{j=1}^{N}\frac{1}{{d}_{ij}}$$

where N is the size of the network, *d*_*ij*_ is the geodesic length which represents the length of the shortest path between node *i* and node *j*. We track the product of average inverse geodesic length of network A and network B, which is computed as:

5 $$\begin{array}{rcl}{{\rm{R}}}_{rl} & = & \frac{1}{{\rm{N}}}\sum _{p=1/N}^{1}{f}_{rl}(p)\\  & = & \frac{1}{{\rm{N}}}\sum _{p=1/N}^{1}{L}_{A}(p)\cdot {L}_{B}(p)\end{array}$$

where *L*_*A*_(*p*), *L*_*B*_(*p*) is the average inverse geodesic length computed by equation (4), and p is the fraction of recovered nodes. It is suitable to measure the condition of coupled networks by ${f}_{r{\rm{l}}}(p)$ because it takes the robustness of the whole coupled network into consideration.

**Figure 3:**
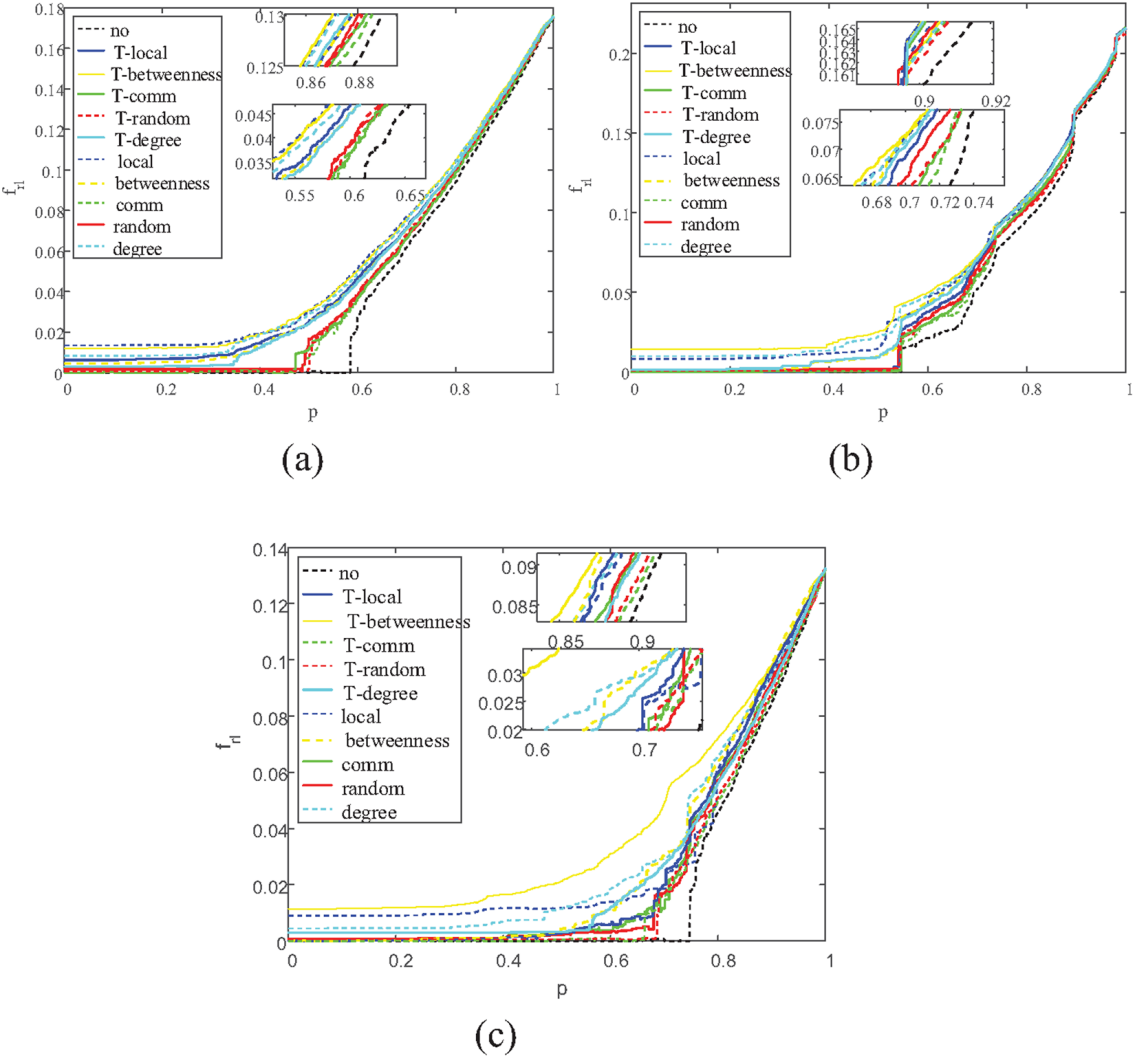
Illustration of the average inverse geodesic length in each iteration $({f}_{rc}(p))$. When fraction p of nodes are recovered. Curves of different color represent different protect strategies, and dashed lines represent the method of single-layer protection respect to a specific strategy while solid lines are their two-layer version. (**a**) Correlation between ${f}_{rl}(p)$ and recovered nodes p on ER-ER system. (**b**) Correlation between ${f}_{rl}(p)$ and recovered nodes p on SF-ER system. (**c**) Correlation between ${f}_{rl}(p)$ and recovered nodes p on Power-ER system.

The results of ${R}_{r{\rm{l}}}$ on three kinds of coupled networks are shown in Table 3 and as it shows, the robustness of the tested coupled networks are enhanced. For example, under the two-layer betweenness strategy, the ${R}_{r{\rm{l}}}$ can reach 0.0584 from 0.0418 on the ER-SF network, comparing to 0.051 under the single method. Performances of the two-layer degree and the two-layer local strategies are not commendable because these two centralities are not focused on the flow of information in networks. Simulations of ${R}_{r{\rm{l}}}$ are shown in Fig. 3. In the diagram the variation of average inverse geodesic length (${f}_{r{\rm{l}}}(p)$) is tracked so that we can see how ${R}_{r{\rm{l}}}$ changes in the recovery process.

**Table 3:** Comparisons of *R*_*rl*_ between existing methods and two-layer protection based on five strategies.

	No	Degree	Local	Betweenness	Comm	Random
ER-ER						
single-layer	0.0394	0.0489	0.0507	0.055	0.0435	0.0445
two-layer	0.0394	0.0531	0.0555	0.0497	0.0434	0.0439
ER-SF						
single-layer	0.0418	0.0509	0.048	0.0584	0.0463	0.0469
two-layer	0.0418	0.0559	0.0542	0.051	0.0456	0.0458
SF-Power						
single-layer	0.0217	0.0259	0.0251	0.0383	0.024	0.0235
two-layer	0.0217	0.0305	0.0278	0.0264	0.0231	0.0229

**Figure 4:**
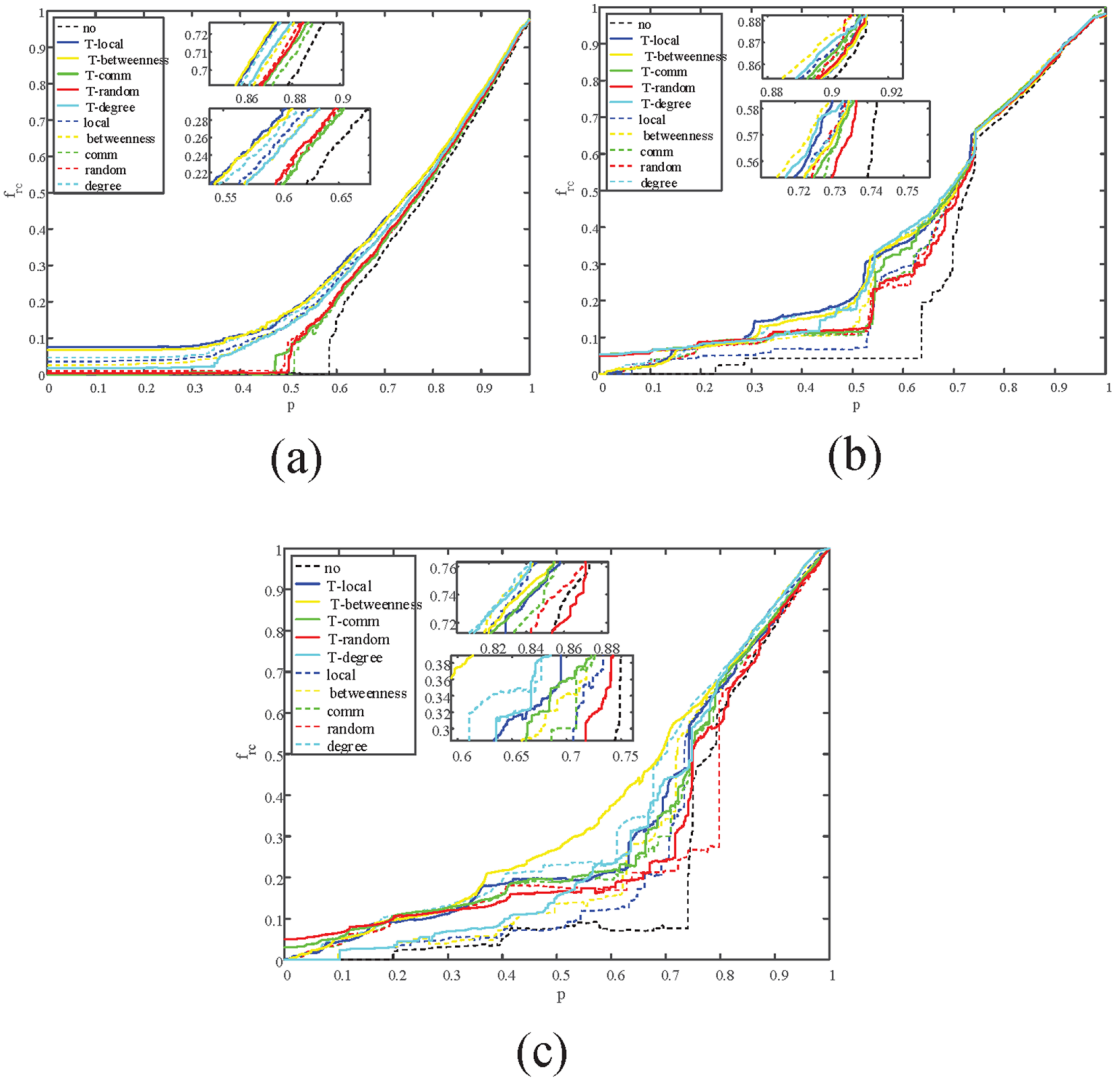
Illustration of the remaining fraction of nodes in each iteration $({f}_{rc}(p))$ when $\lambda$ = **0.3**. (**a**) Correlation between ${f}_{rc}(p)$ and recovered nodes p on ER-ER system. (**b**) Correlation between ${f}_{rc}(p)$ and recovered nodes p on SF-ER system. (**c**) Correlation between ${f}_{rc}(p)$ and recovered nodes p on Power-ER system.

**Figure 5:**
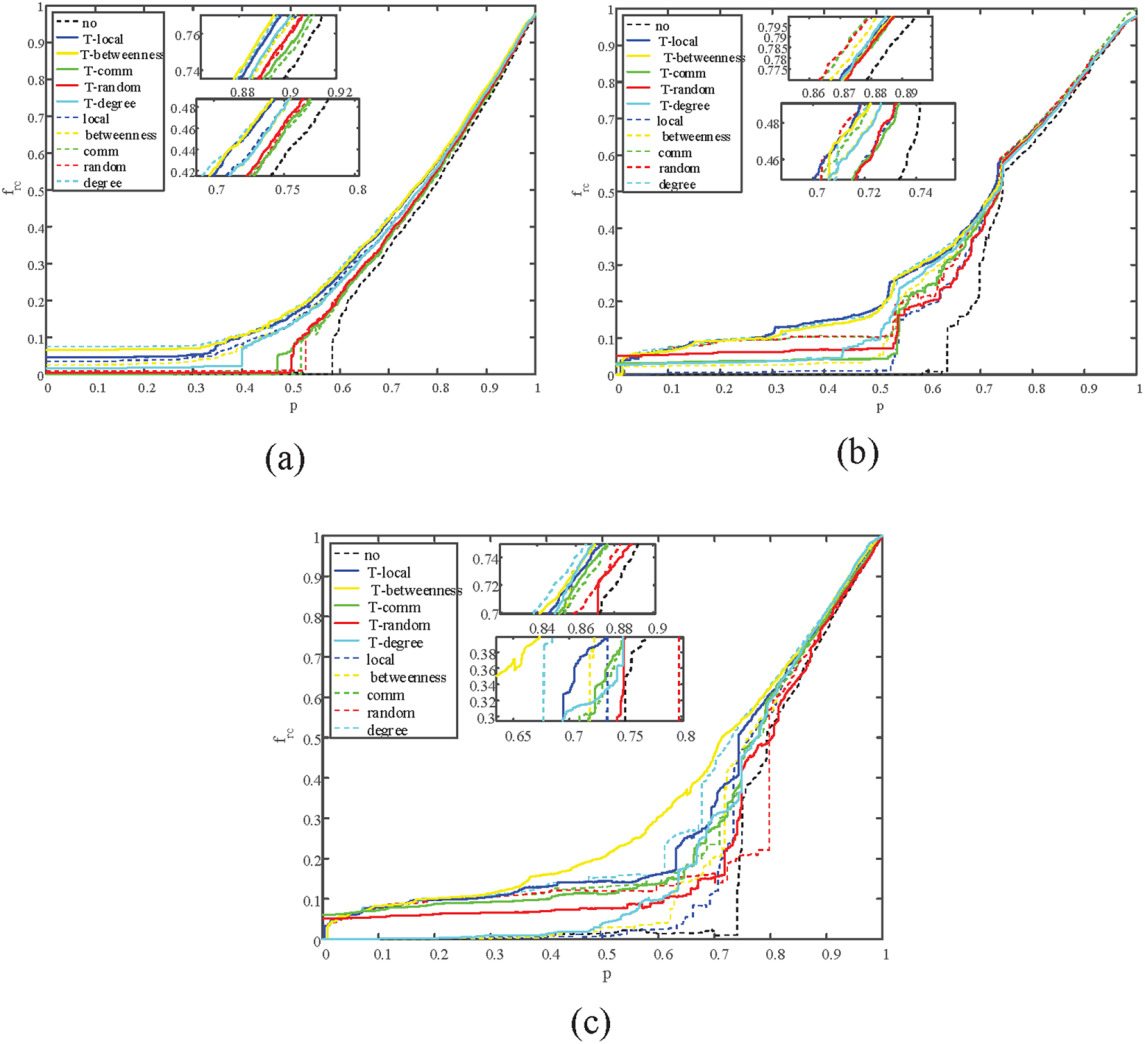
Illustration of the remaining fraction of nodes in each iteration $({f}_{rc}(p))$ when $\lambda$ = **0.8**. (**a**) Correlation between ${f}_{rc}(p)$ and recovered nodes p on ER-ER system. (**b**) Correlation between ${f}_{rc}(p)$ and recovered nodes p on SF-ER system. (**c**) Correlation between ${f}_{rc}(p)$ and recovered nodes p on Power-ER system.

**Figure 6:**
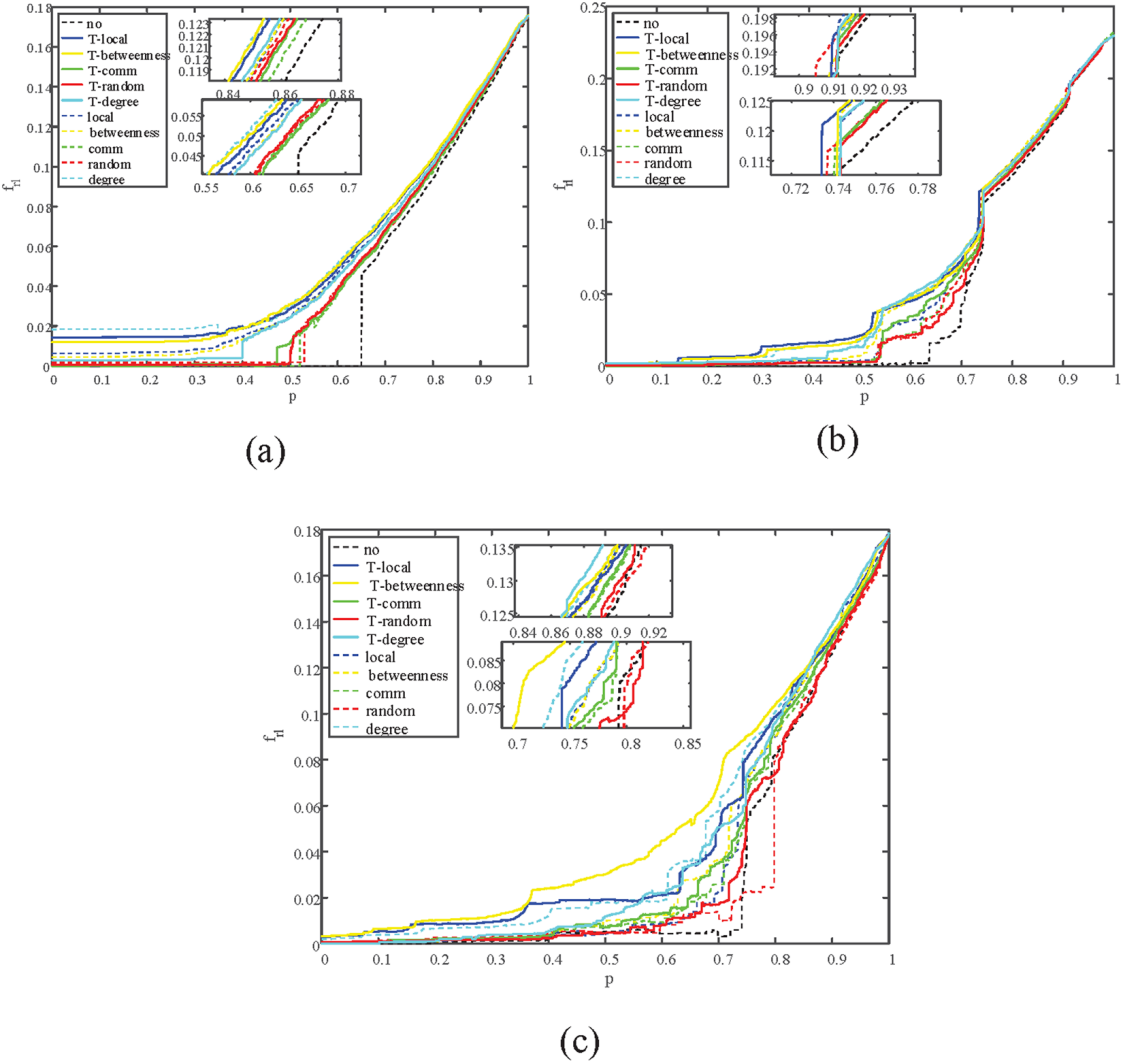
Illustration of the average inverse geodesic length in each iteration $({f}_{rc}(p))$ when $\lambda$ = **0.3**. (**a**) Correlation between ${f}_{rc}(p)$ and recovered nodes p on ER-ER system. (**b**) Correlation between ${f}_{rc}(p)$ and recovered nodes p on SF-ER system. (**c**) Correlation between ${f}_{rl}(p)$ and recovered nodes p on Power-ER system.

**Figure 7:**
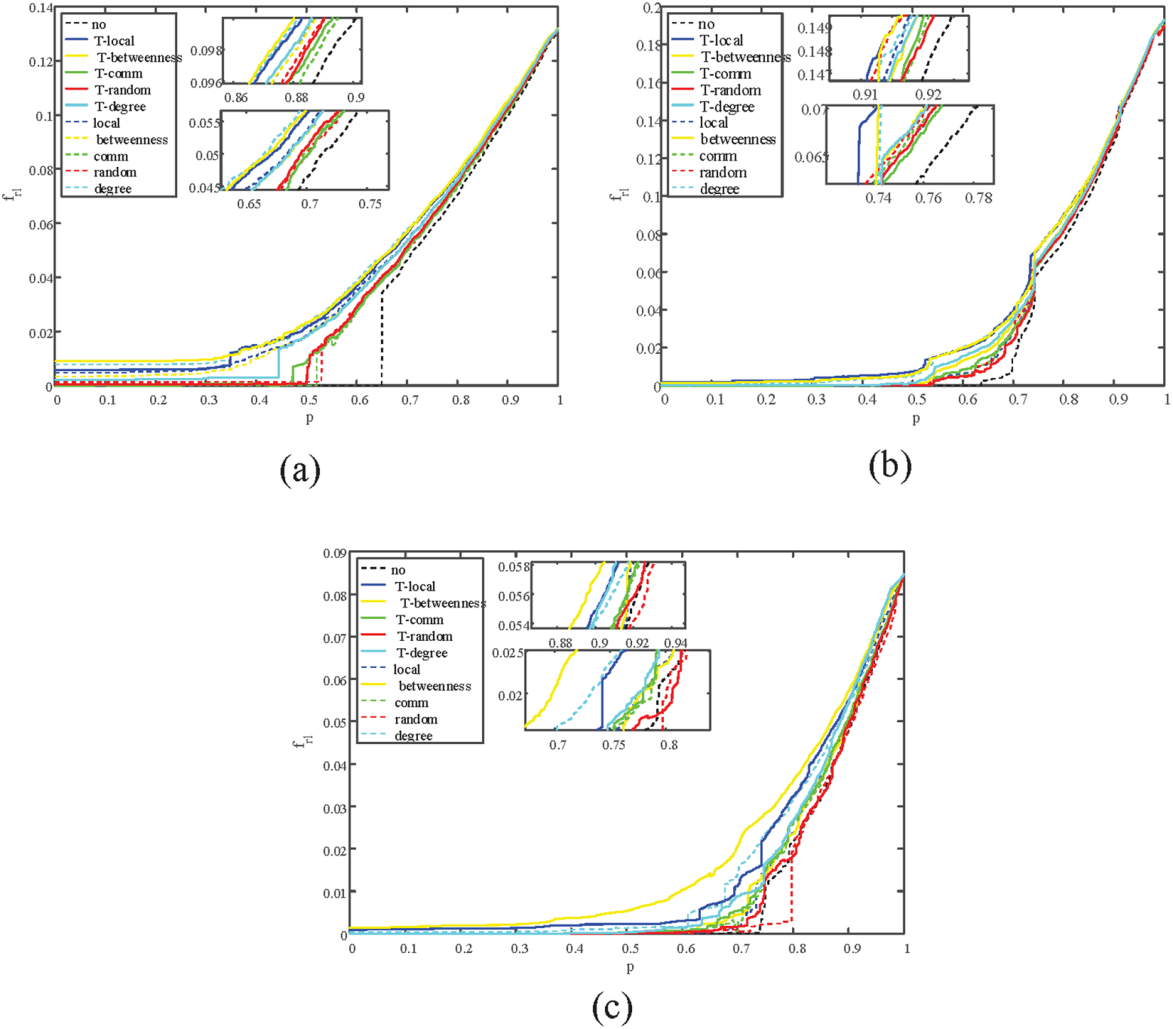
Illustration of the average inverse geodesic length in each iteration $({f}_{rc}(p))$ when $\lambda$ = **0.8**. (**a**) Correlation between ${f}_{rl}(p)$ and recovered nodes p on ER-ER system. (**b**) Correlation between ${f}_{rl}(p)$ and recovered nodes p on SF-ER system. (**c**) Correlation between ${f}_{rl}(p)$ and recovered nodes p on Power-ER system.

To analyze how is*λ* affects the performance of the proposed method, the variation of three kinds of coupled networks with different *λ* are tracked. Results are shown in Figs 4–7. From the diagrams we can conclude that, when *λ* varies, the proposed two-layer method can still recover a coupled network efficiently. Changes of *λ* only affects the absolute value of ${f}_{r{\rm{l}}}(p)$ and ${f}_{r{\rm{c}}}(p)$, and the trend of the variation stays the same.

## Discussion

To sum up, we proposed a two-layer-protection strategy to protect influential nodes in coupled networks based on five different strategies (both global and local) and keep them functional from cascade failures under their recoveries. When global structures are considered, we use the two-layer vision of Degree, Betweenness and Random centralities to select influential nodes. As for local structures, the Comm centrality is introduced into coupled networks to protect nodes. In order to carefully analyze the variations on the fraction of largest connected component and the average inverse geodesic length, the proposed strategy is carried out on the recovery process. Experimental results have shown that, by protecting some influential nodes, the robustness of coupled systems can be enhanced, and the improvement can be more significant under the two-layer method. We also find that, comparing to the single method, the robustness can be greatly improved even for random-connected networks like Erdő-Rényi using the two-layer protection. It is notable that while here the Degree, Betweenness, Local, Comm and Random strategies are used as metrics for two-layer protection, it is feasible that other criterion will also work.

## Methods

### Nodes recovery

As well known, coupled networks are more fragile than single networks when they suffered failures. They are also vulnerable when being recovered. In the real world, coupled networks’ break-down always starts from only one of its networks and then the failure spreads to its coupled network^30^. For example, damages on power station would lead to disconnection of communication network, which will cause a cascade failure in the coupled networks in turn^14^. As in the recovery process, for example, a system coupled by network A and network B could walk through the following process. Firstly, a fraction *p* of nodes in network A(B) are revived, which will trigger the recovery of their coupled nodes in network B(A). The newly recovered nodes in network A(B) that are not in the largest cluster would lose their functionality, and this failure in network A(B) will cause the failure of its coupled nodes in network B(A). This failing process recursively occurs until there are no more failures in both networks.

### Protect influential nodes

Based on the network knowledge, we know that by protecting influential nodes, the robustness of complex networks can be greatly enhanced^15,17,36^. Earlier studies have shown some effective strategies to choose influential nodes.

### Random C_r_(i)

To obtain the influence of nodes, in this strategy, the random centrality of node *i* is computed as:

6 $${C}_{r}(i)=random()$$

where random() is a function to generate a float number between 0 and 1.

### Degree C_d_(i)

In the degree centrality, nodes with high degree are regarded as influential. The centrality is computed as^22^:

7 $${C}_{d}(i)=\sum _{j=1}^{N}{d}_{ij}{a}_{ij}$$

where *d*_*ij*_ is the connection between node *i* and node *j* in the adjacent matrix, in which ${a}_{ij}=1$ represents that there is a connection between node *i* and *j*, otherwise ${a}_{ij}=0$.

### Betweenness C_b_(i)

In this strategy, influence of nodes are measured by the shortest path which passes through the node *i*. The betweenness centrality is calculated as^22^:

8 $${C}_{b}(i)=\sum _{j\ne j,q\ne i}^{N}\frac{{\delta }_{jq}(i)}{{\delta }_{j{\rm{q}}}}$$

where ${\delta }_{jq}$ represents the number of shortest paths from node *j* to node *q*, and ${\delta }_{jq}(i)$ denotes the number of shortest paths walking through node *i* from node *j* to *q*.

### Local C_l_(i)

In local centrality, the standard of measuring the influence of nodes is determined by its direct neighbor and its next nearest neighbor. The Local centrality can be computed as^24^:

9 $${C}_{l}(i)=\sum _{{V}_{j}\in {{\rm{\Phi }}}_{i}}\sum _{{V}_{u}\in {{\rm{\Phi }}}_{j}}m(u)$$

where *m*(*u*) is the number of nodes whose shortest paths from them to node u are less than 3, and Φ_*i*_(Φ_*j*_) is the neighbor of node *i*(*j*).

### Comm C_c_(i)

In order to make sure the proposed two-layer protection is also efficient on networks with modular structure, we adopt Comm strategy in the experiment. The two-layer version of Comm centrality will be discussed in next section as a contrast. This measure takes into account of both intra and inter-community links of a node^37^. When selecting influential nodes, the “hub” and the “bridge” are considered. The “hub” represents a kind of nodes which has many connections with the other nodes in its own community. And the “bridge” is the kind of nodes which connect their community to other communities in the network. The Comm centrality is computed as^37^:

10 $$\begin{array}{rcl}{C}_{c}(i) & = & (1+{\mu }_{com})\ast (\frac{{k}_{i}^{in}}{\mathop{\max ({k}_{j}^{in}\forall j\in C)}\limits_{j}}\ast M)\\  &  & +(1-{\mu }_{com})\,\ast \,{(\frac{{k}_{i}^{out}}{\mathop{\max }\limits_{j}({k}_{j}^{in}\forall j\in C)}\ast M)}^{2}\end{array}$$

where M is an integer number, in order to make sure that both in-degree and out-degree are in the same range. The in-degree ${k}_{i}^{in}$ represents the number of connections connecting it to the nodes of the same community. And the out-degree is equal to the number of edges connecting to other nodes which are outside the community. The in-degree and out-degree are calculated as

11 $${k}_{i}^{in}=\sum _{j\in C}A(i,j),{k}_{i}^{out}=\sum _{j\in C}A(i,j)$$

In the equation (10), ${\mu }_{com}$ represents the fraction of the out-degree to the total connection in community C. The value of ${\mu }_{com}$ can be calculated as

12 $${\mu }_{com}=\frac{\sum _{i\in C}\,{k}_{i}^{out}/{k}_{i}}{size(C)}$$

### Two-layer protection

Coupled networks can be modeled as *G* = (*A, B, L*_*AB*_), where *L*_*AB*_ represents the coupling links between network A and B. In our strategy, each node in network A is randomly coupled with a node of network B. Nodes in network B can be triggered to operate normally if their coupled nodes in network A have been recovered. And nodes are revived gradually with a targeted recover method. Earlier studies have shown that by decoupling nodes^15^, generating autonomous nodes^17^, or by protecting influential nodes^22,23^ can greatly enhance the robustness of coupled networks. But in the real world, for example, in epidemiology, diseases can spread within any populations but can also be transferred other populations, even to different species. In the process of the transmission of disease, one population and another population are both infectious and have equal probability to infect others^38^. Based on the above knowledge, it is reasonable to protect nodes which are important to both layers in the coupled networks rather than only one of them. The proposed strategy consists the following parts: in the beginning, the coupled system *G* = (*A, B, L*_*AB*_) are expended to *G* = (*C*(*L*_*A*_, *L*_*B*_, *L*_*AB*_)), where *L*_*A*_ represents the edges of network A and *L*_*B*_ denotes the edges in network B while *L*_*AB*_ represents the coupling links between network A and B. The initial networks A and B consists N nodes respectively. And the combined network C consists of 2 N nodes. Edges of C are composed of *L*_*A*_, *L*_*B*_ and *L*_*AB*_, where *L*_*AB*_ now are links in network C instead of the coupling links between the two networks. Then a fraction of nodes is selected to be protected in the order of a specific strategy. Based on the phenomena, we propose a two-layer-protection technique for five strategies respectively. With this strategy, the robustness of coupling networks can be greatly enhanced. Strategies used to protect influential nodes are given as follows.

### T-degree(two-layer-degree)

**Figure 8:**
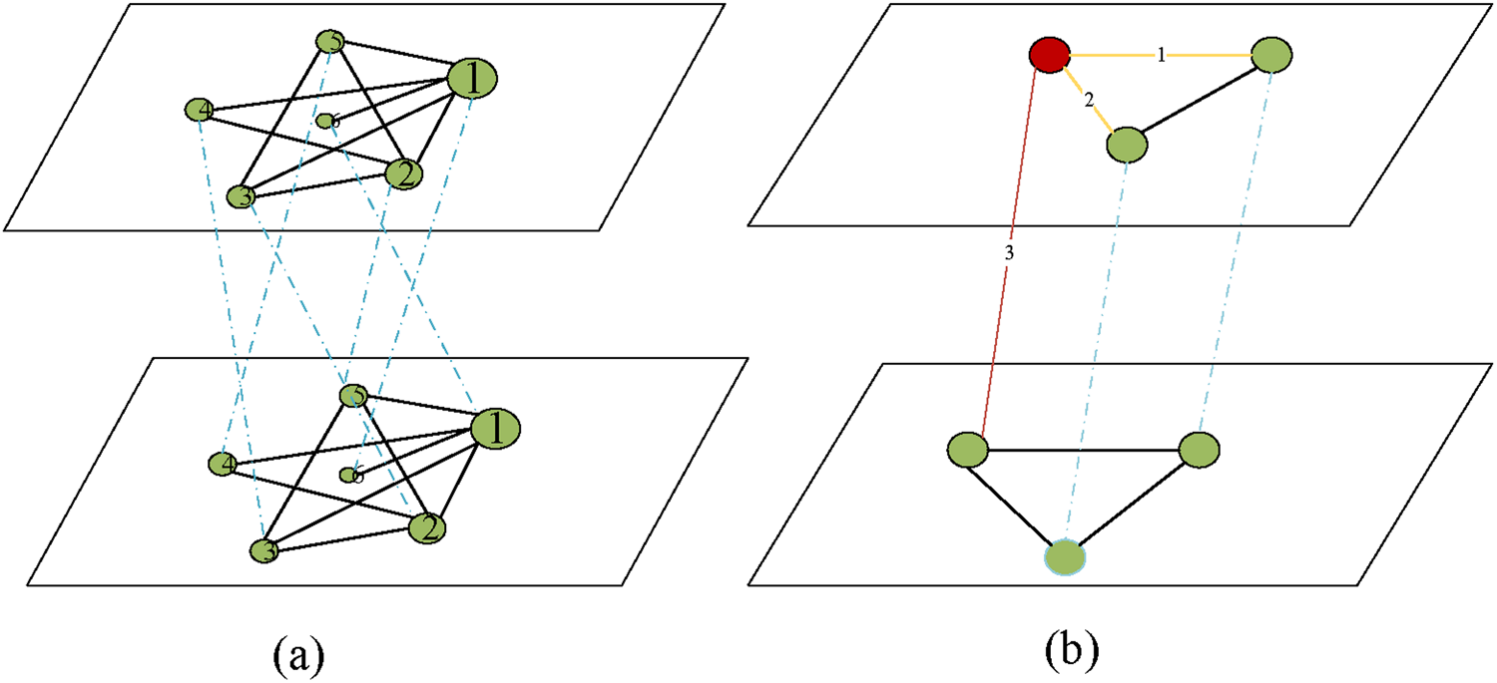
Illustration of proposed protecting model on a small coupled network. (**a**) Illustration of T-degree model, in which nodes are listed in descending order of their degree. (**b**) Illustration of T-betweenness model. In the betweenness centrality model, when the protection is limited to one layer, the node *i* (marked in red) only controls the information flow in link 1, 2. In the proposed two layer model, node *i* controls the information flow in link 1, 3 additionally.

In the T-degree strategy, nodes are selected to be influential in the descending order of the degree centrality. For example, a toy system coupled by network A and B are given in Fig. 8(a). In the small system, 2 nodes are under protection. In the traditional way, node 1 and 2 in network A are chosen so that they and their coupled nodes can operate normally when failure occurs. By doing so, there is a node with degree 5 and a node with degree 4 under protection in the system. Experiment shows that, with the fraction of protected nodes remaining unchanged, protecting node 1 in network A and node 1 in network B lead to a better performance.

### T-betweenness (two-layer-betweenness)

Firstly the coupled system *G* = (*A, B, L*_*AB*_) are extended to *G* = (*C*, (*L*_*A*_, *L*_*B*_, *L*_*AB*_)), where *L*_*A*_ represents the edges of network A and *L*_*B*_ denotes the edges in network B while *L*_*AB*_ represents the coupling links between network A and B. The initial networks A(B) consists N nodes respectively. And the combined network C consists of 2 N nodes. Edges of C are composed of *L*_*A*_, *L*_*B*_ and *L*_*AB*_, where *L*_*AB*_ now are normal links in network C instead of the coupling links between the two networks. Secondly we search the whole network C and select a fraction of nodes which have the highest betweenness and protect them from damages. Nodes in network C are ranked in a descend order according to the betweenness centrality, which can be calculated as equation (8). By combining network A and B into one, the betweenness of one node $i$ now represents the number of the shortest paths in network C that pass through *i*, which means the paths arch across the system form network A to B are also considered. These crossing paths play important roles in information delivery and failure spreading, but they are ignored in the traditional studies. Illustration is given in Fig. 8(b).

### T-comm (two-layer-comm)

**Figure 9:**
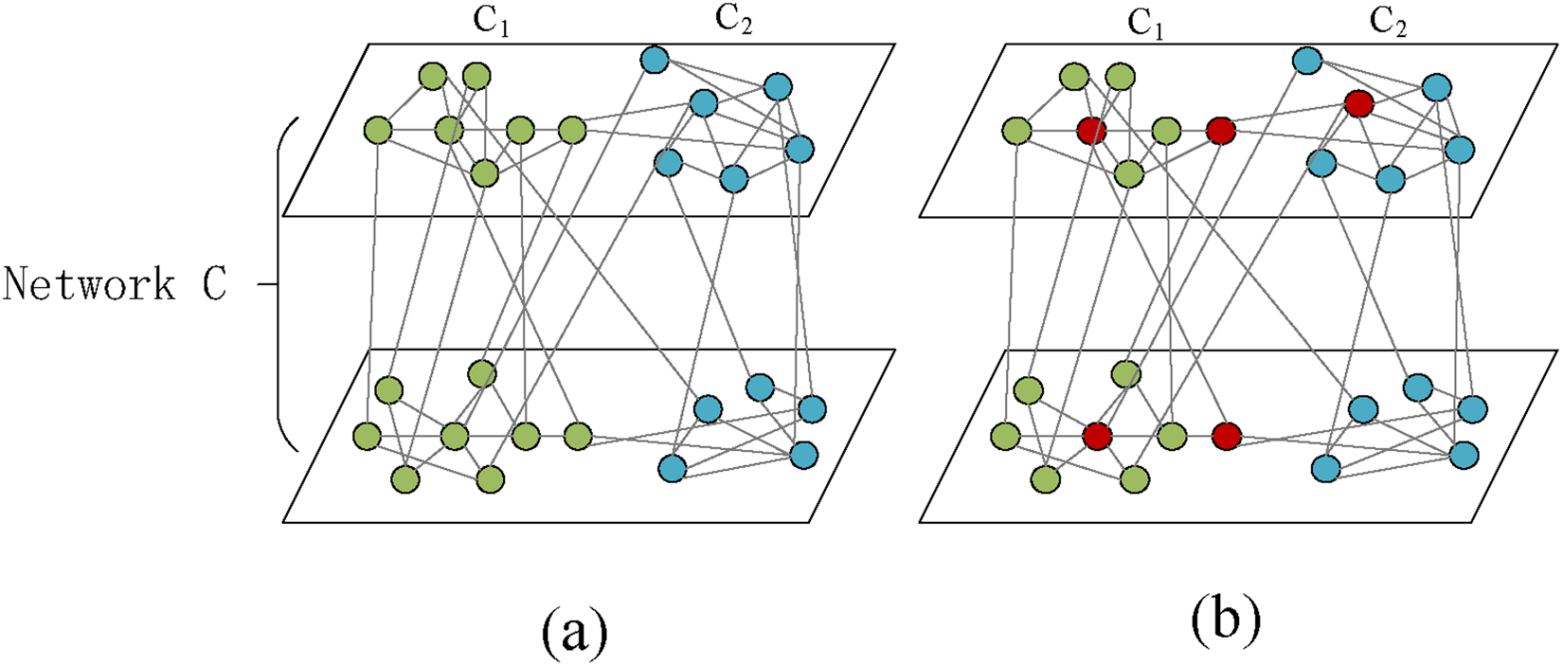
Illustration of proposed model on a small coupled network. (**a**) The community distribution in network C. Community ${C}_{1}{C}_{2}$ are marked in green and blue respectively. (**b**) Nodes selected by the TLCP model are highlighted in red.

It is good to explain the strategy by giving an example. When some recovered nodes of network A in community C_1_ lose their functions, nodes which are directly connected with them would fail. Then this failure would affect other nodes in community C_1_, which would cause a complete fragmentation to the whole community C_1_. Nodes in network B which are coupled with the nodes in community C_1_ would fail, and in this situation, it might lead to a further fragmentation in network B. It is good to explain the proposed strategy by giving an example. In the following illustration, influence nodes are selected in the order of their comm centralities (equation (10)). In order to obtain the community property, a community detection process is needed. But detecting communities in network A and B respectively is not enough because nodes in a small community in layer 1 could be coupled with nodes in a large community in layer 2. In this case, the communities in network A(B) cannot represent the structure of the system. We extend the work in refs^37,39^ by protecting influential nodes selected based on the T-comm centrality, the performance of coupled networks in the failing procedure is improved. To detect communities in both layers and regard them as a single network, firstly we expend the system *G* = (*A, B, L*_*AB*_) to *G* = (*C*,(*L*_*A*_, *L*_*B*_, *L*_*AB*_)). Then a community detection procedure is implemented on network C. Here we use the BGLL model^39^. There are two steps in this model. (1) Every nodes in network C is assigned a community, and each node *i* is assigned to the community of its neighbor *j*, if the gain of modularity ${\rm{\Delta }}Q$ is maximum in this process. This step stops when a local maximum of the modularity is attained. (2) Regard each community which is obtained in step (1) as a “node” and repeat the procedure until the modularity stops changing. The gain of modularity ${\rm{\Delta }}Q$ by moving an isolated node i into community C can be computed as

13 $${\rm{\Delta }}Q=[\frac{{\sum }_{in}\,+\,{k}_{i,in}}{2m}-{(\frac{\sum _{tot}{k}_{i}}{2m})}^{2}]-[\frac{{\Sigma }_{in}}{2m}-{(\frac{{\Sigma }_{tot}}{2m})}^{2}-{(\frac{{k}_{i}}{2m})}^{2}]$$

where ${{\rm{\Sigma }}}_{in}$ is the sum of the weights of the links that are inside community C, ${{\rm{\Sigma }}}_{tot}$ represents the sum of the weights of the links which link to nodes in community C, *k*_*i*_ is the degree of node *i*, *k*_*i,in*_ is the sum of the weights of links between *i* and nodes in community C, and *m* represents the sum of weights of links in the entire network. In our experiment, all weights are set to be 1 because the networks we use are all undirected. Then the Comm centrality of the combined network C is calculated. Then a small fraction of nodes which have the highest Comm centrality are protected. Therefore, this small fraction of nodes can operate normally when they or their coupled nodes suffer from damages. An instance of community distribution is given in Fig. 9(a). The selected nodes to be protected are shown in Fig. 9(b). To explain easily, five nodes are protected intuitively in the illustration.

### T-local (two-layer-local)

In this strategy, same procedure is implemented as the above strategies to generate network C. Then we calculate the local centrality as equation (9). Note that in equation (9), neighbors of node *i* now contains not only the nodes directly linked with it but also the node coupled with it in another network. By doing so, the nearest and next nearest nodes which play crucial roles when measuring the influence of node *i* will be distributed in both network A and B, instead of only in one of them. In this way, nodes that make a major contribution to the cascade failure can be captured.
